# Antimycobacterial activity of the plectasin derivative NZ2114

**DOI:** 10.3389/fmicb.2025.1613241

**Published:** 2025-06-26

**Authors:** Camilla Davids, Komal Rao-Fransson, Nitya Krishnan, Erik Tenland, Matthias Mörgelin, Brian Robertson, Gabriela Godaly

**Affiliations:** ^1^Department of Microbiology, Immunology and Glycobiology, Institution of Laboratory Medicine, Lund University, Lund, Sweden; ^2^Centre for Bacterial Resistance Biology, Department of Infectious Disease, Imperial College London, London, United Kingdom; ^3^Department of Clinical Immunology and Transfusion Medicine, Skåne University Hospital, Lund, Sweden; ^4^Colzyx AB, Lund, Sweden

**Keywords:** *Mycobacterium tuberculosis*, tuberculosis, peptides, plectasin, treatment

## Abstract

**Introduction:**

Mycobacteria have a unique hydrophobic membrane with several lipid-enriched layers that are low in permeability, setting them apart from other bacteria. This complex structure, consisting of three distinct layers is crucial for cell growth, virulence, and providing a barrier to antibiotics. Previously, we identified a plectasin variant, NZX, which showed activity against *Mycobacterium tuberculosis* in several murine tuberculosis (TB) infection studies. In this study, we investigated another plectasin variant, NZ2114, known for its effectiveness against Gram-positive bacteria, as a potential antimycobacterial peptide both *in vitro* and *in vivo*.

**Methods:**

The resazurin microtiter assay (REMA) was used to determine MIC; a time-kill assay was performed to evaluate long-term effects; scanning electron microscopy (SEM) was employed to visualize peptide impact; a checkerboard assay assessed drug compatibility; MTT and WST-8 assays were used to estimate peptide toxicity; intracellular killing was evaluated using primary macrophages; peptide stability was assessed in human serum; and a murine tuberculosis (TB) infection model was used to verify the peptide’s efficacy.

**Results:**

NZ2114 effectively killed mycobacteria at a minimal inhibitory concentration (MIC_99_) of 6.1 µM, was non-toxic to primary human cells, and remained resistant to serum degradation while preserving its antimycobacterial capacity. In a checkerboard assay, NZ2114 demonstrated synergy with the first-line TB drugs isoniazid and ethambutol. The antimicrobial effect was also observed against several clinical isolates of Gram-positive bacteria, including *Enterococcus faecalis, Enterococcus faecium*, and Methicillin-Resistant *Staphylococcus aureus* (MRSA). In our murine TB infection model, compared to untreated controls, NZ2114 eliminated *M. tuberculosis* with a log reduction of 0.72 (81.14%) after three doses.

**Discussion:**

These studies suggest NZ2114 as a potential TB therapy, aiding in the control of this significant infectious disease.

## Introduction

Antimicrobial resistance (AMR) is a global concern that affects public health and poses a significant threat to worldwide development. In 2021, bacterial AMR was estimated to have contributed to 4.71 million deaths, with projections suggesting this number could rise to 8.22 million by 2050 (GBD 2021 Antimicrobial Resistance Collaborators, 2024). The World Health Organization (WHO) recently published a bacterial priority pathogens list, categorizing pathogens into critical, high, and medium priority groups (WHO, 2024a). Rifampicin-resistant *Mycobacterium tuberculosis* (RR-Mtb) is now included in the critical group, with tuberculosis (TB), among the top 10 causes of mortality worldwide. In 2020, RR-Mtb was responsible for 6.93 million disability-adjusted life years (DALYs), primarily due to morbidity and mortality during treatment (Menzies et al., 2023). Additionally, TB often results in long-term morbidity among survivors. The significance of the WHO’s list is to highlight the urgent need for new antimicrobial agents against these highly resistant bacteria.

Since their discovery in the 1980s, there has been extensive research to develop antimicrobial peptides (AMPs) for treating microbes, including drug-resistant bacteria. AMPs are small, naturally occurring molecules that play a crucial role in the innate immune system by directly killing a wide range of pathogens, including bacteria, viruses, and fungi. As of January 2025, the Antimicrobial Peptide Database (APD) records a total of 5,099 AMPs (College of Medicine Nebraska University, 2025). Among the thousands of AMPs identified, plectasin and its variants, isolated from the fungus *Pseudoplectania nigrella*, are notable. These peptides are known to kill Gram-positive bacteria, including *Staphylococcus aureus* and its methicillin-resistant variant (MRSA), by inhibiting bacterial cell wall biosynthesis (Mygind et al., 2005; Schneider et al., 2010). NZ2114, is one of the plectasin variants, and has shown improved efficacy against Gram-positive bacteria in models of CNS infection and experimental endocarditis (Ostergaard et al., 2009; Xiong et al., 2011).

TB remains a significant global health challenge, causing over 1.5 million deaths annually (WHO, 2024b). The cell wall of *M. tuberculosis* is highly complex and robust, providing a formidable barrier against many antimicrobial agents. This complexity includes a thick, waxy layer of mycolic acids that makes the bacterium particularly resistant to many conventional antibiotics (Daffe and Marrakchi, 2019). However, some AMPs can penetrate this barrier and exert their antimicrobial effects (Sonawane et al., 2011; Rivas-Santiago et al., 2005; Tenland et al., 2018). In this study, we evaluated the efficacy of NZ2114 in killing mycobacterial strains and clinical isolates of several Gram-positive bacteria and assessed the combination treatment of NZ2114 and first-line TB drugs on mycobacterial clearance. The peptide’s biocompatibility, serum half-life activity, and intracellular capacity were investigated using mycobacteria and human primary macrophages, and the antimicrobial capacity of NZ2114 was further analyzed in a murine TB infection model, where three doses of NZ2114 significantly reduced bacterial loads.

## Methods

### Peptide

The peptide NZ2114 (GFGCNGPWNEDDLRCHNHCKSIKGYKKKYCAKGGFVCKC) was manufactured by solid phase peptide synthesis, followed by cyclisation of three natural occurring di-sulphide bonds and purification by sequential chromatography steps (PolyPeptide Laboratories AB, Limhamn, Sweden). The purity (>97%) of the peptide was confirmed by high-performance liquid chromatography. The peptide has a molecular weight of 4,411 Da and net charge +4.6 at pH 5.5 (Boge et al., 2018).

### Bacteria

For MIC analysis and checkerboard experiments, *Mycobacterium bovis* Bacillus Calmette–Guérin (BCG) Montreal containing the pSMT1-*luxAB* plasmid was utilized (Snewin et al., 1999). Briefly, BCG was grown in Middlebrook 7H9 broth, supplemented with 10% ADC enrichment (Middlebrook Albumin Dextrose Catalyse supplement, Becton Dickinson, Oxford, United Kingdom) and hygromycin (50 mg/L; Roche, Lewes, United Kingdom) (Tenland et al., 2019). Before each experiment, a vial was defrosted, added to 9 mL of 7H9/ADC/hygromycin medium and placed in the shaking incubator for 7 days at 37°C. The bacterial bioluminescence Relative Light Units (RLU) were quantified by adding 0.1% decanal and measuring RLU in a luminometer (TrisStar, Berthhold Technologies, Germany) (Tenland et al., 2018).

For the checkerboard experiments, *Mycobacterium bovis* Bacillus Calmette–Guérin (BCG) (ATCC) was used and cultured as above, without hygromycin. An optical density (OD₆₀₀) of 0.01 corresponds to approximately 10^6^ CFU/mL, as routinely verified by plating and colony counting (CFUs). Additionally, for screening experiments, three or more clinical isolates of each of the following bacteria were used: *Mycobacterium abscessus* and the Gram-positive *Enterococcus faecalis*, *Enterococcus faecium*, methicillin resistant *Staphylococcus aureus* (MRSA), *Staphylococcus aureus* and *Streptococcus pneumoniae*. These isolates were obtained from Clinical Microbiology, Regional Laboratories Skåne, Lund, Sweden, and used for MIC analysis. The Gram-positive strains were grown in LB-broth, overnight in the 37°C shaking incubator, while the isolates of *M. abscessus* were grown as described above for BCG. The mycobacterial strains and the Gram-positive bacteria were quantified using the spectrophotometer and reading the optical density (OD) at 600 nm.

### Minimum inhibitory concentration

For minimum inhibitory concentration (MIC) experiments, we used the resazurin microtiter assay (REMA) as previously published (Rao et al., 2021). Briefly, the bacteria were seeded equally at an OD of 0.01 (~10^6^ CFU/mL) in 96-well plates and incubated at 37°C and 5% CO_2_ with NZ2114 at concentrations ranging from 25 μM to 0.2 μM using a two-fold dilution series. Treatment time was set according to individual strain doubling time. Prestoblue cell viability reagent (Thermo Scientific) was added to the untreated control to monitor the growth process of the bacteria. 1:10 and 1:100 dilutions were used as additional growth controls (Rao et al., 2021). MIC was determined as the concentration where no colour change was observed (i.e., the lowest concentration that prevents visible growth of the bacteria), while the controls had turned from blue to pink indicating growth. The reported MICs are all 99% inhibition.

### Time-kill assay

The time-kill assay was conducted as previously described (Tenland et al., 2018). *Mycobacterium bovis* BCG was cultured to the logarithmic growth phase (approximately 10^3^ CFU/mL) and exposed to AP2114 at final concentrations of 0.4, 0.8, 1.6, and 3.2 μM. Duplicate samples were collected daily from both treated and untreated control cultures. To each sample, 0.1% (v/v) n-decyl aldehyde (Decanal; Sigma-Aldrich) was added, and bioluminescence was measured as relative light units (RLU) over a 1-s integration time using a TriStar^2^ microplate reader (Berthold Technologies). The data shown are representative of two independent biological replicates.

### Scanning electron microscopy

The impact of the peptide on *M. bovis* BCG was assessed using scanning electron microscopy (SEM). The bacteria were cultivated to a concentration of 1 × 10^8^ CFU and subsequently treated with 6.3 μM of the peptide for a duration of 0 or 24 h. Following treatment, the bacteria were concentrated by centrifugation at 3,000 × g for 7 min, re-suspended in a fixation solution (comprising 4% formaldehyde and 2.5% glutaraldehyde in sodium cacodylate), and then deposited onto poly-L-lysine-coated glass coverslips for a duration of 1 h. The samples were processed according to previously established protocols (Svensson et al., 2014) and analyzed using a Philips/FEI XL30 FEG scanning electron microscope (Philips, Lund, Sweden) at an acceleration voltage of 5 kV and a working distance of 10 mm.

### Cell culture

Human macrophages were isolated following a previously published protocol in which monocytes were obtained from healthy volunteers using a lymphoprep density gradient medium (Axis-Shield, Oslo, Norway) as previously published (Tenland et al., 2018). CD14 microbeads were added to the cell suspension, washed, and passed through a LS-column (Miltenyi Biotec) to produce pure monocytes. The monocytes were counted using the Sysmex and diluted in RPMI 1640, supplemented with 5% FCS, NEAA, 1 mM Sodium Pyruvate, 0.1 mg/mL Gentamicin and 50 ng/mL M-CSF and finally seeded in 96-well plates for 7 days to differentiate into macrophages (Tenland et al., 2018).

### Cytotoxicity

To measure the biocompatibility of the NZ2114 peptide with primary human macrophages, primary macrophages were incubated overnight with NZ2114 at concentrations ranging from 25 μM to 0.2 μM using a two-fold dilution series in fresh RPMI1640 medium. Following overnight incubation at 37°C and 5% CO_2_, MTT (16.5 μL, Sigma) was added to each well and incubated for 1 h at 37°C, and the absorbance was measured on a plate reader at 535 nm. For the WST-8 cytotoxicity test (Abcam), NZ2114 treated primary macrophages were incubated overnight at 37°C and 5% CO_2_. WST-8 solution (10 μL) was then added to each well, and the cells were incubated for 2 h at 37°C whereafter the absorbance was measured at 480 nm.

### Peptide interaction with current TB antibiotics

Drug interactions between NZ2114 and currently used TB drugs including, rifampicin (RIF), isoniazid (INH), ethambutol (EMB), kanamycin (KAN) and amikacin (AMK) were investigated with the checkerboard assay according to previous publication (Rao et al., 2021). Briefly, bacterial suspension (80 μL) was exposed to 10 μL of each drug [RIF (0.002–0.06 μg/mL), INH (0.03–2 μg/mL), EMB (0.06–4 μg/mL), KAN (0.116–20.1 μg/mL), AMK (0.02–1 μg/mL 2.1–6.82 μM) with NZ2114 (0.88–110 μg/mL)] to test for synergy on a 96-well plate. Living bacteria were detected using resazurin and MTT assays. Resazurin was added to the wells and incubated 24 h at 37°C and 5% CO_2_. For plates analysed by MTT, 10 μL of MTT (1:10 v/v, sigma) was added and incubated overnight at 37°C and 5% CO_2_. Fractional inhibitory concentration (FIC) was calculated based on the MICs of individual drugs and their combinations, against BCG, using three replicates. The FIC index was then calculated using the following equation: ΣFIC = FIC_A_ + FIC_B_ = (C_A_/MIC_A_) + (C_B_/MIC_B_), where MIC_A_ and MIC_B_ are the MICs of drugs A and B when tested individually, and CA and CB are the concentrations of the drugs in combination, taken from representative wells showing no bacterial growth (Rao et al., 2021).

### Intracellular killing

The prepared primary human macrophages were used to measure intracellular MIC as previously published with some modification (Tenland et al., 2018). Briefly, BCG was added to the macrophages at a multiplicity of infection (MOI) of 10:1 and incubated for 24 h at 37°C, 5% CO_2_. After a 4-h infection at 37°C in 5% CO_2_, macrophages were treated with 200 mg/L amikacin for 30 min at 37°C, 5% CO_2_, washed twice with DMEM to eliminate extracellular bacteria. The cells were washed three times with PBS, replaced with DMEM medium (Thermo Fisher) and treated with NZ2114 at concentrations ranging from 0.2–12.5 μM. Rifampicin was used as a positive control at a concentration of 0.1 μg/mL (0.7 μM) (Schon et al., 2009). The cells were then incubated at 37°C, 5% CO_2_ for 6 days. To determine the intracellular killing capacity, macrophages were lysed with sterile water (30 min) and incubated with presto blue cell reagent (Thermo Fisher) for 24 h at 37°C, 5% CO_2_. The following day the fluorescence intensity was measured at 620 nm.

### Human serum stability

Serum stability studies we performed as previously described (Rao et al., 2021). NZ2114 was incubated in human serum at concentrations ranging from 0.2–12.5 μM for 1, 2 and 3 h, at 37°C, 5% CO_2_. Serum was used to prepare the serial dilutions of NZ2114. After each time, 10 μL of serum-incubated NZ2114 was added to 90 μL of BCG suspension (OD 0.01) and incubated at 37°C, 5% CO_2_ for 7 days. Serum incubated rifampicin was used as a control at a concentration of 0.1 μg/mL (0.7 μM) (Schon et al., 2009). PrestoBlue was added to the cells and incubated overnight at 37°C, 5%CO_2_. The following day the fluorescence intensity was measured at 620 nm.

### Murine TB infection model

All animal procedures were conducted under a license issued by the UK Home Office, in compliance with the Animal Scientific Procedures Act of 1986. Female BALB/c mice, aged 6 to 8 weeks (Charles River Ltd., United Kingdom), were housed in biosafety level 3 (BSL3) facilities at Imperial College London, following institutional protocols (Marquina-Castillo et al., 2009). Animals were kept in groups of five per cage under standard conditions, with ad libitum access to food and water. Mice were monitored daily for general activity and grooming behaviour. Body weight was recorded weekly, and a humane endpoint was defined as a weight loss of ≥18% or the appearance of other clinical signs of illness. Veterinary oversight was available throughout the study. The mice were infected with approximately 5 × 10^3^ CFU/mL of *M. tuberculosis* H37Rv via the intranasal route. The control group consisted of eight mice, including three used to check bacterial implantation in the lungs on day 2. One group of five mice was treated with three doses for 1 week with 33 mg/kg NZ2114, administered intranasally in 35 μL PBS. The control group received 35 μL PBS via the same route. Following treatment, the mice were euthanized, and their lungs were aseptically removed. The lung tissue was homogenized in PBS with 0.05% Tween-80, serially diluted, and plated on Middlebrook 7H11 agar plates supplemented with 0.5% glycerol and 10% OADC. Colony-forming units (CFU) were counted 21 days later.

### Statistical analysis

Statistics was generated using the Prism software (version 10). One-way ANOVA for multiple comparisons followed by the *post-hoc* test was used to calculate significance for the serum incubation and checkerboard experiments. Significance was accepted at ^*^p < 0.05, ^**^p < 0.01 or ^***^p < 0.001.

### Ethical statement

All animal procedures were performed under the license issued by the UK Home Office and in accordance with the Animal Scientific Procedures Act of 1986. The animal studies have been approved by the Local Animal Welfare and Ethical Review Board (London, UK) (Numbers PPL 70/7160 and 70/8653). The blood for monocyte isolation for the toxicity experiments was donated by healthy volunteers (Local Ethical Review Board Dnr 2011/403 and 2014/35). The healthy volunteers were provided with verbal and written information about the study’s purpose, duration, potential risks and benefits. Personal data was not collected from the volunteers, and the blood was pooled for the isolation of monocytes.

## Results

### Antimicrobial activities of NZ2114

**Figure 1:**
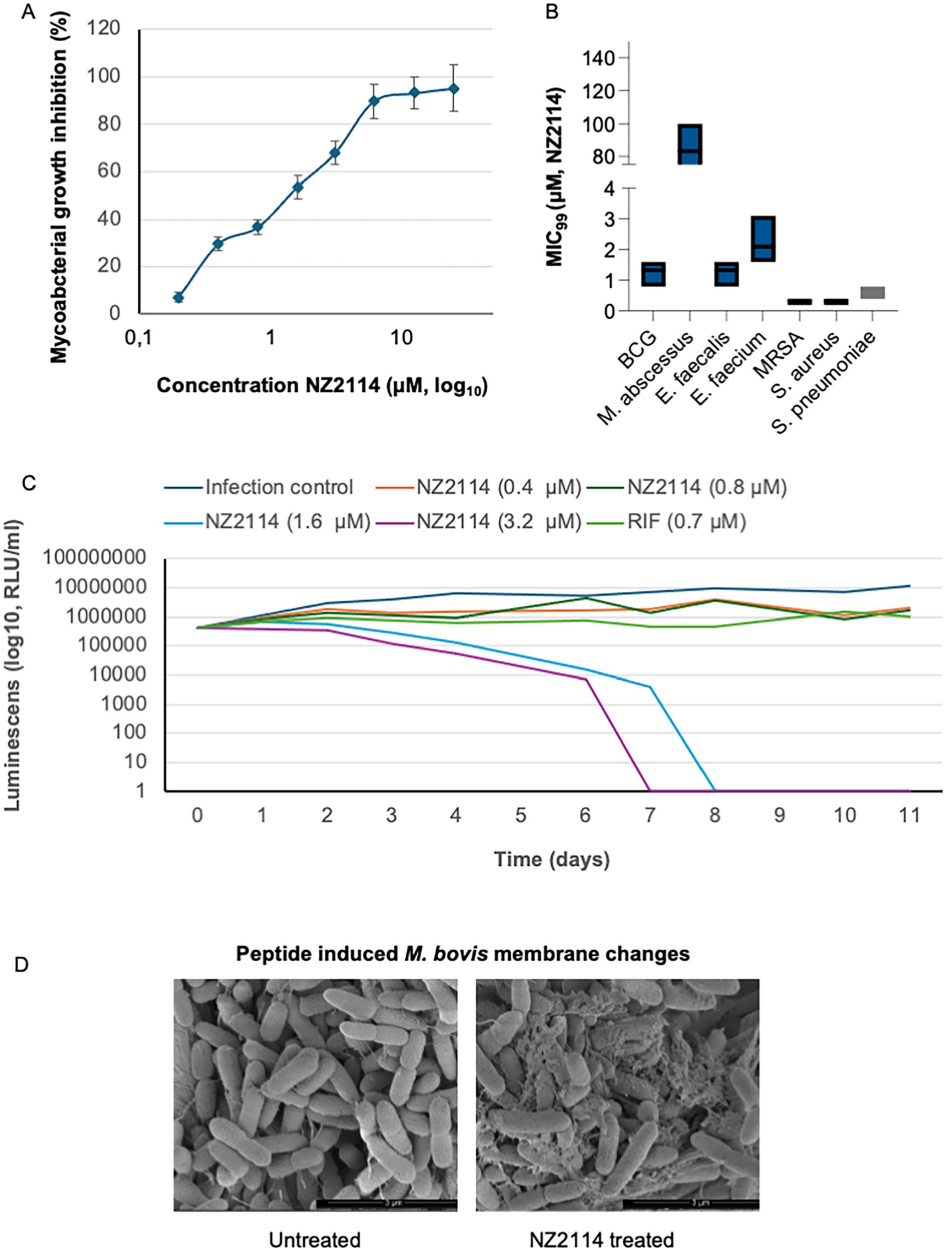
Antimicrobial activity of NZ2114. **(A)** The peptide showed concentration-dependent mycobacterial inhibition towards BCG, with a MIC concentration of 6.1 μM. For *M. abscessus*, the MIC value was significantly higher, averaging 75 μM. Bacterial growth inhibition is shown as percentage of untreated bacteria. **(B)** Among the Gram-positive isolates, vancomycin-resistant *E. faecium* exhibited the highest inhibitory concentration, while the MIC_99_ for *E. faecalis* was the same as for BCG. Methicillin-resistant *S. aureus* and methicillin-susceptible *S. aureus* had the lowest MIC_99_ values, followed by *S. pneumoniae*. Treatment time was set according to individual strain doubling time. *n* = 3 for all bacterial strains tested. **(C)** Time-kill assay. At day 0, the BCG was treated once with 0.4, 0.8, 1.6 or 3.2 μM of NZ2114. Luminescence was measured twice per time point. **(D)** BCG was treated with 6.3 μM NZ2114 for 24 h and visualized by scanning electron microscopy (SEM). Membrane destabilization was observed. Scale bar: 3 μm. Experiment was performed in triplicate.

NZ2114 was previously demonstrated to have increased inhibitory activity against Gram-positive bacteria compared to plectasin (Ostergaard et al., 2009). In this study, we tested this peptide for antimicrobial activity against mycobacteria and a broad range of Gram-positive pathogens. NZ2114 exhibited potent activity towards two strains of *M. bovis BCG*, which belong to the *M. tuberculosis* complex, as well as clinical isolates of *M. abscessus* and several clinical isolates of Gram-positive bacteria (Figures 1A,B). For BCG, there was a concentration-dependent inhibition, with a MIC_90_ concentration of 6.1 μM (Figure 1A). For *M. abscessus*, the MIC_99_ value was much higher, with a mean of 75 μM. For the Gram-positive isolates, the vancomycin-resistant *E. faecium* had the highest inhibitory concentration at 6.1 μM, while the MIC_99_ of *E. faecalis* was the same as for BCG (Figure 1B). The methicillin-resistant *S. aureus* and methicillin-susceptible *S. aureus* had the lowest MIC_99_ value at 0.3 μM, followed by *S. pneumoniae* with an inhibitory concentration of 0.5 μM of NZ2114. The time-kill assay showed that a single dose of NZ2114 at 1.6 μM or 3.2 μM eliminated BCG by day 8 and day 7, respectively (Figure 1C). The assay also indicated that low NZ2114 concentrations possess bactericidal activity.

### Peptide interaction with *Mycobacterium bovis* BCG membrane

In our previous investigations into the mechanisms of the NZX peptide, we found that it exhibited strong affinity for the mycobacterial membrane (Rao et al., 2023). In this study, we treated *M. bovis* BCG with 6.3 μM of the related peptide NZ2114. Untreated bacteria appeared as smooth, rod-shaped cells (Figure 1D). In contrast, peptide-treated bacteria exhibited membranous protrusions and extensive bubbling in all cells.

### NZ2114 is not toxic to human cells

**Figure 2:**
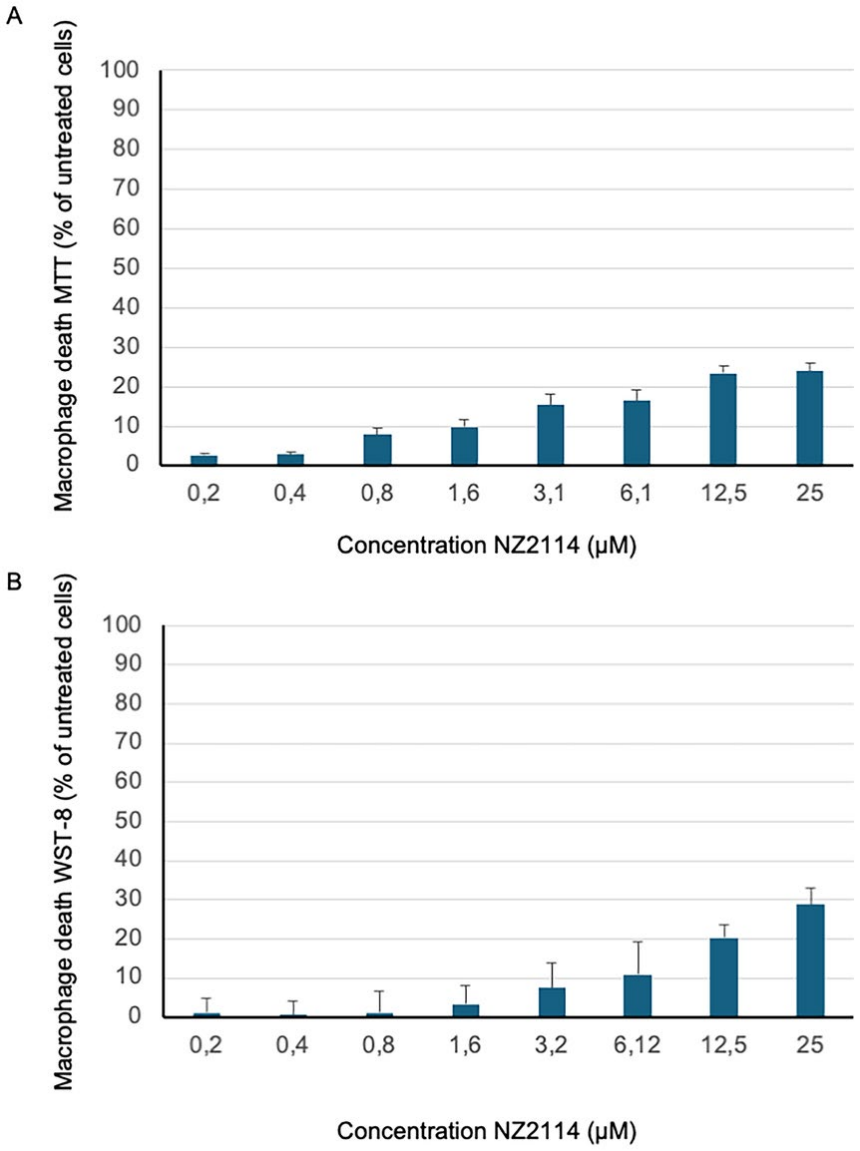
NZ2114 toxicity analysis. The cytotoxicity of NZ2114-treated primary macrophages was assessed using **(A)** the MTT cell viability assay and **(B)** the WST-8 assay, with results expressed as a percentage of the untreated control. Both assays demonstrated that 24 h of NZ2114 incubation did not exhibit any toxic effects on the cells at therapeutic concentrations. No significant reduction in cell viability was observed at these concentrations. *n* = 3.

To evaluate the cytotoxicity of NZ2114, two different assays were employed the MTT assay, and the WST-8 assay. These assays were conducted using human primary macrophages, which were prepared from whole blood samples (see above). The results from both assays indicated that NZ2114 did not exhibit any toxic effects on the cells at concentrations up to 25 μM (LD50 >25 μM). This was confirmed by the data presented in Figures 2A,B, where no significant reduction in cell viability was observed at these concentrations.

### NZ2114 induces intracellular killing of *Mycobacterium bovis* BCG

**Figure 3:**
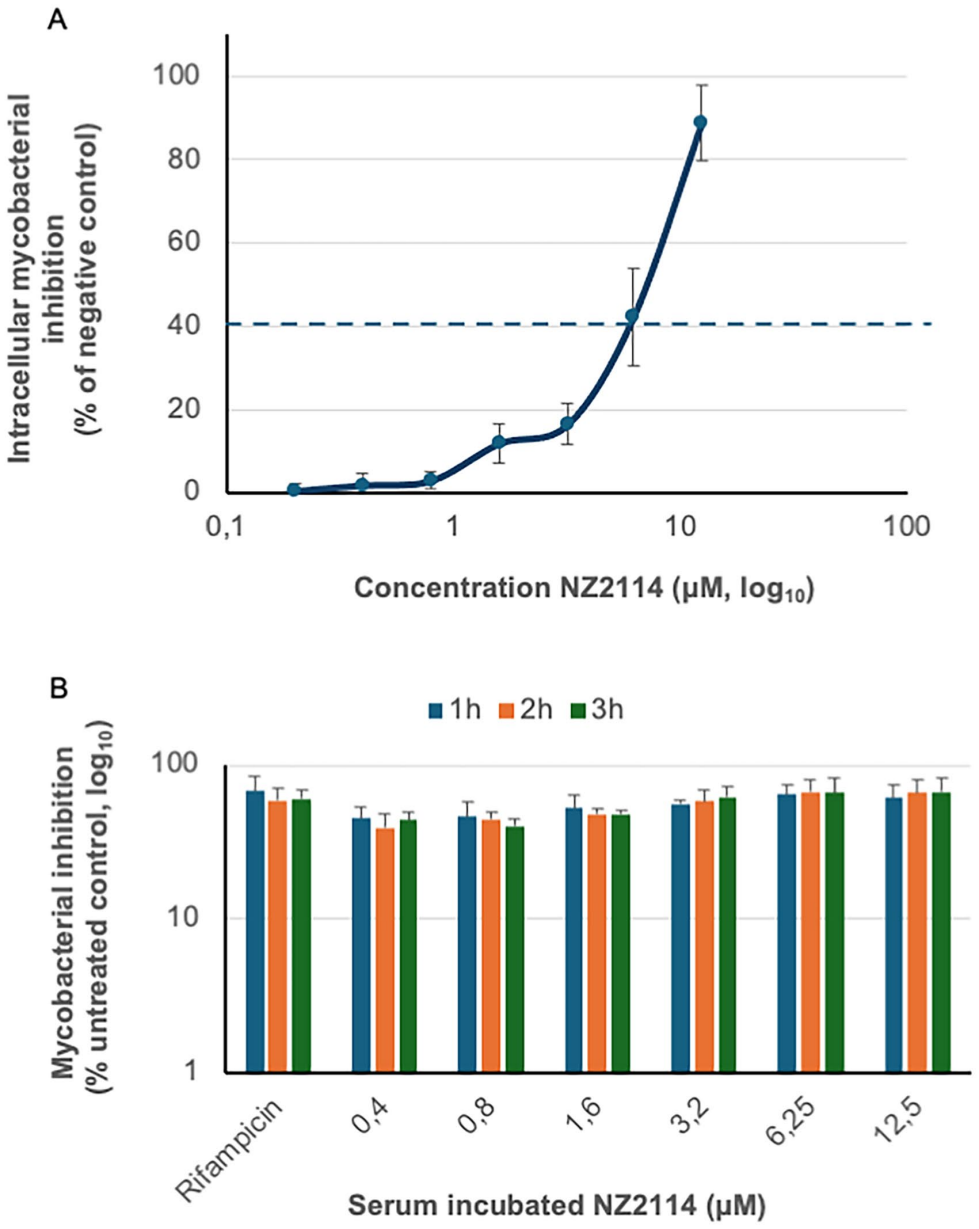
Therapeutic analysis of NZ2114. **(A)** The intracellular anti-mycobacterial capacity of NZ2114 was evaluated using primary human macrophages infected with BCG. One dose of NZ2114 demonstrated a concentration-dependent reduction in the intracellular bacterial load after 6 days, with rifampicin serving as a positive control (concentration of 0.1 μg/mL, dotted line). Bacterial growth inhibition is shown as percentage of untreated bacteria. **(B)** The serum resistance of NZ2114 was assessed by incubating the peptide in human serum for up to 3 h, followed by determining its function against mycobacteria. Rifampicin, used as a control at a concentration of 0.7 μM, also preserved its antimycobacterial capacity after same serum incubation. No statistical difference was found between the different NZ2114 concentrations or the rifampicin. *n* = 3.

The intracellular anti-mycobacterial capacity of NZ2114 was assessed after 6 days using primary human macrophages infected with BCG. NZ2114 demonstrated a concentration-dependent reduction in the intracellular bacterial load, achieving a maximum reduction of 89% at a concentration of 12.5 μM (Figure 3A). Rifampicin, used as a positive control at a concentration of 0.1 μg/mL (0.7 μM), resulted in a similar level of intracellular bacterial killing as observed with 6.12 μM of NZ2114 (dotted line).

### Serum incubation did not alter peptide efficacy

Intravenous therapy is essential for treating severe mycobacterial infections. Previous studies have shown that plectasin has a terminal serum elimination half-life of 51 min (Mygind et al., 2005). In our investigation, we analysed the impact of serum on the function of NZ2114 by determining the MIC value (Figure 3B). Our results indicated that the MIC values remained stable, with remained antimycobacterial capacity, for up to 3 h of serum incubation. Rifampicin, a first-line anti-tuberculosis drug known for its stability in serum, was used as a control. At a concentration of 0.7 μM, rifampicin also preserved its antimycobacterial capacity after same serum incubation. There was no statistical difference between the different concentration or the rifampicin.

### NZ2114 exhibited a synergistic effect with current TB drugs

**Figure 4:**
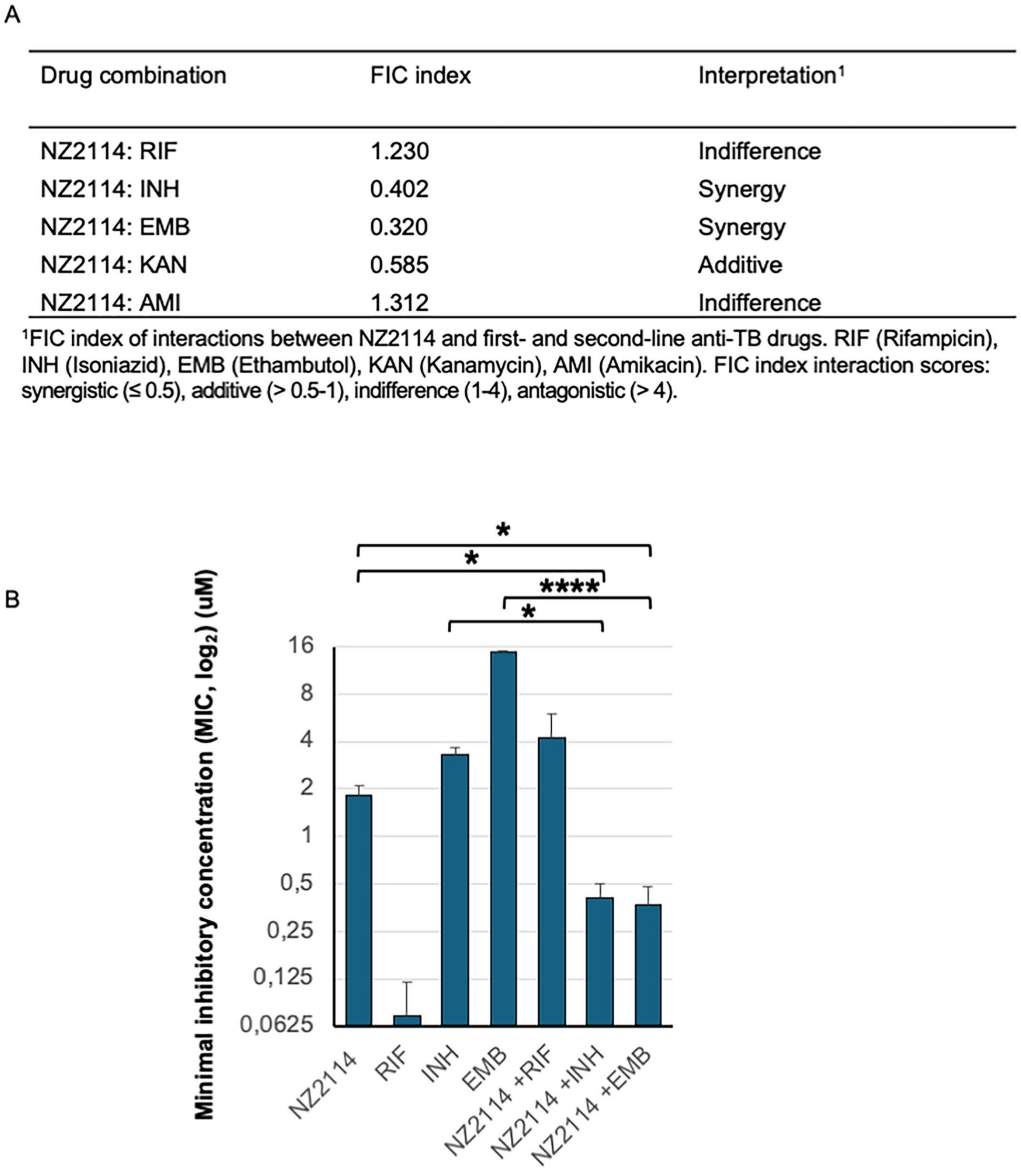
NZ2114 interactions with current TB drugs. **(A)** The FIC index of interactions in mycobacterial elimination between NZ2114 and the TB drugs rifampicin, isoniazid, ethambutol, amikacin, and kanamycin was analysed using checkerboard assays and the MTT assay. **(B)** A graph illustrating mycobacterial elimination 24 h after treatment with rifampicin (RIF), isoniazid (INH), ethambutol (EMB), or NZ2114 alone, or in combination with NZ2114, showed a synergistic effect. The experiments were repeated three times. One-way ANOVA for multiple comparisons followed by the *post-hoc* test was used to calculate accepted significance, ^*^p < 0.05 and ^****^p < 0.0001.

The interactions between NZ2114 and the TB drugs rifampicin, isoniazid, ethambutol, amikacin and kanamycin were analysed using checkerboard assays and the MTT assay (Supplementary Table 2). The FIC index analysis revealed that the interactions between NZ2114 and these TB drugs were predominantly synergy or additive/indifferent, meaning there were no significant changes to their respective independent MIC values. Importantly, none of the drug combinations demonstrated antagonistic effects. However, it was noteworthy that NZ2114 exhibited a synergistic effect when combined with ethambutol (EMB) and isoniazid (INH), as evidenced by the lower MIC values observed in these combinations (Figure 4).

### Efficacy of NZ2114 against *Mycobacterium tuberculosis* in a murine infection model

**Figure 5:**
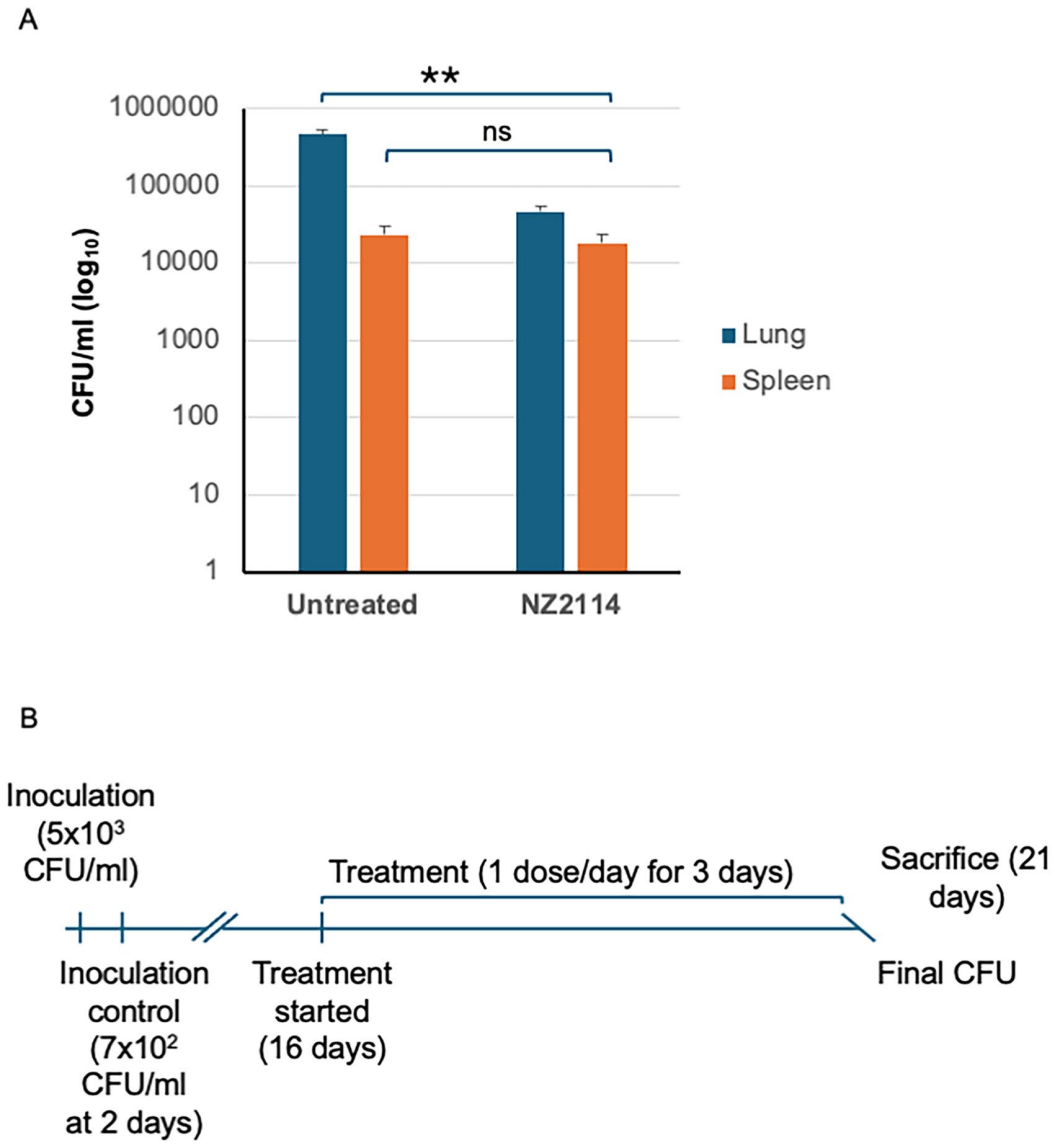
*In vivo* evaluation of NZ2114. **(A)** A schematic of experimental setup for murine pulmonary TB with *M. tuberculosis* H37Rv strain. **(B)** Three doses of NZ2114 administered endotracheally over 1 week reduced lung CFU with a log reduction of 0.72 (81%) on day 21. Data are presented as mean ± SD. *p*-value was calculated by Mann–Whitney test (^**^p = 0.0079).

To validate our drug interaction results, we conducted an experiment using a murine model of infection with the *M. tuberculosis* H37Rv strain. The mean bacterial implantation dose in the lungs, measured 2 days post-infection, was 700 CFU/lung. After 21 days, when treatment commenced, the bacterial load in the untreated control group had increased to 3.835 × 10^7^ CFU. All untreated mice survived the entire duration of the experiment. In the treated NZ2114-treated, a general reduction in CFU of 1.001 log₁₀ was observed compared to the untreated mice (*p* = 0.0079) (Figure 5). These results demonstrate the efficacy of NZ2114 in reducing the bacterial load in a murine infection model, supporting the potential use of this peptide for TB therapy.

## Discussion

In this study, we investigated the potential of NZ2114 in the treatment of mycobacterial infections. We found this peptide to hold key attributes for future therapy, such as its ability to induce mycobacterial killing at low minimum inhibitory concentrations and its effective elimination of *M. tuberculosis* in our murine TB model. Comparing the MIC values from other antimycobacterial peptides with similar molecular weights, such as beta-defensin (HBD)-1, HBD-2, and LL-37, summarized in Jacobo-Delgado et al. (2023), shows that NZ2114 shows low antimycobacterial MIC values which is important for possible future TB therapy. Furthermore, we found that a single dose of NZ2114 to BCG induced a long-term inhibitory effect affecting mycobacterial survival. Interestingly, another plectasin derivative, NZX, was previously shown to be bactericidal in its ability to kill both drug-sensitive and drug-resistant mycobacteria belonging to the *M. tuberculosis* complex, such as *M. tuberculosis* and BCG, but also against nontuberculous mycobacteria (NTM) (Tenland et al., 2018; Rao et al., 2021). Interestingly, comparing NZ2114 with NZX, there is a four amino acid difference at positions N9S, L13I, R14Q, and K32R, but none of these belong to the crucial amino acids to retain peptide *S. aureus* activity (Schneider et al., 2010). Further studies also reviled that plectasin targeted bacterial cell wall precursor lipid II to eliminate *S. aureus* (Schneider et al., 2010; Jekhmane et al., 2024). Previously, we investigated the mechanisms by which NZX eliminates mycobacteria, which possess a more complex outer envelope compared to Gram-positive bacteria (Rao et al., 2023). NZX was found to interact with the mycobacterial inner membrane and to target multiple essential enzymes involved in mycolic acid synthesis that could impact bacterial proliferation (Rao et al., 2023). In this study, we observed that NZ2114 treatment caused disruption of the mycobacterial membrane; however, the underlying mechanism requires further investigation. The *M. abscessus* complex comprises rapidly growing, multidrug-resistant NTM that can lead to pulmonary disease, particularly in vulnerable individuals with underlying structural lung conditions like cystic fibrosis, bronchiectasis, and previous TB (Griffith et al., 2007). When comparing the effectiveness of NZ2114 and NZX against *M. abscessus*, NZX proved to be more potent, eliminating the pathogen more efficiently at lower concentrations (Rao et al., 2021). Plectasin, NZ2114 and NZX share possibly structural similarity as they have disulfide bonds at positions C4–C30, C15–C37, and C19–C39 (Mygind et al., 2005; Rao et al., 2021). Whether structure similarity or subtle differences in amino acids composition affect the peptide’s antimycobacterial capacity is not clear, but these results indicate that NZ2114 is similarly effective against mycobacteria compared to NZX.

Moreover, NZ2114 exhibits broad-spectrum antimicrobial activity, effectively targeting a range of bacterial pathogens, including *E. faecalis*, *E. faecium*, MRSA, *S. aureus*, and *S. pneumoniae*. This broad activity highlights NZ2114’s versatility and potential utility in treating various infections. The drug-resistant (DR) *E. faecium* is included in the high-risk WHO category (WHO, 2024a). *E. faecium* is typically a benign bacterium that can cause severe infections in vulnerable individuals (Rosselli Del Turco et al., 2021). It shows a concerning resistance to antibiotics, especially vancomycin, posing significant challenges in healthcare facilities. Also found in this group is Methicillin-resistant *S. aureus* (MRSA), which continues to pose a significant global burden. The MIC value of NZ2114 for *E. faecium* is the highest among the bacteria tested here, but still modest at 2.1 μM, and 1.3 μM for *E. faecalis*, fully comparable to vancomycin concentrations used to treat both drug-sensitive and drug-resistant variants of these pathogens (Sahm et al., 1989; Breidenstein et al., 2015). For the Gram-positive *S. aureus* and MRSA, often causing wound infections, the MIC values of NZ2114 were at 0.3 μM, and for pneumonia-causing *S. pneumoniae*, at 0.5 μM, supported by previous studies (Brinch et al., 2010; Horak and Wenthold, 2009). Taken together, NZ2114 shows potential antimicrobial activity against a broad range of clinical isolates of Gram-positive bacteria.

To eliminate intracellular pathogens, such as *M. tuberculosis*, a drug should possess intracellular capacity. Previous studies analysed NZ2114 combination therapies and intracellular capacity in eliminating *S. aureus* (Xiong et al., 2011; Brinch et al., 2010; Zhang et al., 2014). We investigated the mycobacteria-eliminating activity of NZ2114, showing significant intracellular activity and sustained effectiveness even after 3 h of incubation in human serum. Furthermore, we found this peptide to be compatible with existing TB drugs, which is vital for its potential use in combination therapies. The interaction between NZ2114 and ethambutol and isoniazid had a synergistic interaction score, while the interaction score between NZ2114 and kanamycin, rifampicin and amikacin were additive or indifferent. Supporting the therapeutic possibilities of NZ2114 further, toxicology experiments indicated that NZ2114 is less toxic at high concentrations, in comparison to other TB drugs (Copaescu et al., 2021). Finally, *in vivo* studies demonstrated that NZ2114 can significantly reduce *M. tuberculosis* burden in animal models, with an 81% reduction after three doses, supporting its potential as an effective treatment for mycobacterial infections. Future research should focus on clinical *M. tuberculosis* isolates and more NTM strains to confirm these results and to further explore the therapeutic potential of NZ2114. Structural knowledge of whether this peptide also impair cell wall synthesis needs also be investigated.

## Data Availability

The original contributions presented in the study are included in the article/Supplementary material, further inquiries can be directed to the corresponding author.

## References

[ref1] Boge L. Vastberg A. Umerska A. Bysell H. Eriksson J. Edwards K.. (2018). Freeze-dried and re-hydrated liquid crystalline nanoparticles stabilized with disaccharides for drug-delivery of the plectasin derivative AP114 antimicrobial peptide. J. Colloid Interface Sci. 522, 126–135. doi: 10.1016/j.jcis.2018.03.062, PMID: 29587194

[ref2] Breidenstein E. B. Courvalin P. Meziane-Cherif D. (2015). Antimicrobial activity of plectasin NZ2114 in combination with cell wall targeting antibiotics against VanA-type *Enterococcus faecalis*. Microb. Drug Resist. 21, 373–379. doi: 10.1089/mdr.2014.0221, PMID: 25785733

[ref3] Brinch K. S. Tulkens P. M. Van Bambeke F. Frimodt-Moller N. Hoiby N. Kristensen H. H. (2010). Intracellular activity of the peptide antibiotic NZ2114: studies with Staphylococcus aureus and human THP-1 monocytes, and comparison with daptomycin and vancomycin. J. Antimicrob. Chemother. 65, 1720–1724. doi: 10.1093/jac/dkq159, PMID: 20534628

[ref4] College of Medicine Nebraska University (2025). Antimicrobial Peptide Database. Available online at: https://aps.unmc.edu/ (Accessed April 1, 2025).

[ref5] Copaescu A. Choshi P. Pedretti S. Mouhtouris E. Peter J. Trubiano J. A. (2021). Dose dependent antimicrobial cellular cytotoxicity-implications for ex vivo diagnostics. Front. Pharmacol. 12:640012. doi: 10.3389/fphar.2021.640012, PMID: 34447304 PMC8383281

[ref6] Daffe M. Marrakchi H. (2019). Unraveling the structure of the mycobacterial envelope. Microbiol. Spectr. 7:GPP3-0027-2018. doi: 10.1128/microbiolspec.GPP3-0027-2018, PMID: 31267927 PMC10957186

[ref7] GBD 2021 Antimicrobial Resistance Collaborators (2024). Global burden of bacterial antimicrobial resistance 1990–2021: a systematic analysis with forecasts to 2050. Lancet 404, 1199–1226. doi: 10.1016/S0140-6736(24)01867-1, PMID: 39299261 PMC11718157

[ref8] Griffith D. E. Aksamit T. Brown-Elliott B. A. Catanzaro A. Daley C. Gordin F.. (2007). An official ATS/IDSA statement: diagnosis, treatment, and prevention of nontuberculous mycobacterial diseases. Am. J. Respir. Crit. Care Med. 175, 367–416. doi: 10.1164/rccm.200604-571ST, PMID: 17277290

[ref9] Horak M. Wenthold R. J. (2009). Different roles of C-terminal cassettes in the trafficking of full-length NR1 subunits to the cell surface. J. Biol. Chem. 284, 9683–9691. doi: 10.1074/jbc.M807050200, PMID: 19188369 PMC2665089

[ref10] Jacobo-Delgado Y. M. Rodriguez-Carlos A. Serrano C. J. Rivas-Santiago B. (2023). *Mycobacterium tuberculosis* cell-wall and antimicrobial peptides: a mission impossible? Front. Immunol. 14:1194923. doi: 10.3389/fimmu.2023.1194923, PMID: 37266428 PMC10230078

[ref11] Jekhmane S. Derks M. G. N. Maity S. Slingerland C. J. Tehrani K. Medeiros-Silva J.. (2024). Host defence peptide plectasin targets bacterial cell wall precursor lipid II by a calcium-sensitive supramolecular mechanism. Nat. Microbiol. 9, 1778–1791. doi: 10.1038/s41564-024-01696-9, PMID: 38783023 PMC11222147

[ref12] Marquina-Castillo B. Garcia-Garcia L. Ponce-de-Leon A. Jimenez-Corona M. E. Bobadilla-Del Valle M. Cano-Arellano B.. (2009). Virulence, immunopathology and transmissibility of selected strains of *Mycobacterium tuberculosis* in a murine model. Immunology 128, 123–133. doi: 10.1111/j.1365-2567.2008.03004.x, PMID: 19191912 PMC2747145

[ref13] Menzies N. A. Allwood B. W. Dean A. S. Dodd P. J. Houben R. James L. P.. (2023). Global burden of disease due to rifampicin-resistant tuberculosis: a mathematical modeling analysis. Nat. Commun. 14:6182. doi: 10.1038/s41467-023-41937-9, PMID: 37794037 PMC10550952

[ref14] Mygind P. H. Fischer R. L. Schnorr K. M. Hansen M. T. Sonksen C. P. Ludvigsen S.. (2005). Plectasin is a peptide antibiotic with therapeutic potential from a saprophytic fungus. Nature 437, 975–980. doi: 10.1038/nature04051, PMID: 16222292

[ref15] Ostergaard C. Sandvang D. Frimodt-Moller N. Kristensen H. H. (2009). High cerebrospinal fluid (CSF) penetration and potent bactericidal activity in CSF of NZ2114, a novel plectasin variant, during experimental pneumococcal meningitis. Antimicrob. Agents Chemother. 53, 1581–1585. doi: 10.1128/AAC.01202-08, PMID: 19188395 PMC2663087

[ref16] Rao K. U. Henderson D. I. Krishnan N. Puthia M. Glegola-Madejska I. Brive L.. (2021). A broad spectrum anti-bacterial peptide with an adjunct potential for tuberculosis chemotherapy. Sci. Rep. 11:4201. doi: 10.1038/s41598-021-83755-3, PMID: 33603037 PMC7892554

[ref17] Rao K. U. Li P. Welinder C. Tenland E. Gourdon P. Sturegard E.. (2023). Mechanisms of a *Mycobacterium tuberculosis* active peptide. Pharmaceutics 15:540. doi: 10.3390/pharmaceutics15020540, PMID: 36839864 PMC9958537

[ref18] Rivas-Santiago B. Schwander S. K. Sarabia C. Diamond G. Klein-Patel M. E. Hernandez-Pando R.. (2005). Human beta-defensin 2 is expressed and associated with *Mycobacterium tuberculosis* during infection of human alveolar epithelial cells. Infect. Immun. 73, 4505–4511. doi: 10.1128/IAI.73.8.4505-4511.2005, PMID: 16040961 PMC1201238

[ref19] Rosselli Del Turco E. Bartoletti M. Dahl A. Cervera C. Pericas J. M. (2021). How do I manage a patient with enterococcal bacteraemia? Clin. Microbiol. Infect. 27, 364–371. doi: 10.1016/j.cmi.2020.10.029, PMID: 33152537

[ref20] Sahm D. F. Kissinger J. Gilmore M. S. Murray P. R. Mulder R. Solliday J.. (1989). *In vitro* susceptibility studies of vancomycin-resistant *Enterococcus faecalis*. Antimicrob. Agents Chemother. 33, 1588–1591. doi: 10.1128/AAC.33.9.1588, PMID: 2554802 PMC172707

[ref21] Schneider T. Kruse T. Wimmer R. Wiedemann I. Sass V. Pag U.. (2010). Plectasin, a fungal defensin, targets the bacterial cell wall precursor lipid II. Science 328, 1168–1172. doi: 10.1126/science.1185723, PMID: 20508130

[ref22] Schon T. Jureen P. Giske C. G. Chryssanthou E. Sturegard E. Werngren J.. (2009). Evaluation of wild-type MIC distributions as a tool for determination of clinical breakpoints for *Mycobacterium tuberculosis*. J. Antimicrob. Chemother. 64, 786–793. doi: 10.1093/jac/dkp262, PMID: 19633001

[ref23] Snewin V. A. Gares M. P. Gaora P. O. Hasan Z. Brown I. N. Young D. B. (1999). Assessment of immunity to mycobacterial infection with luciferase reporter constructs. Infect. Immun. 67, 4586–4593. doi: 10.1128/IAI.67.9.4586-4593.1999, PMID: 10456904 PMC96782

[ref24] Sonawane A. Santos J. C. Mishra B. B. Jena P. Progida C. Sorensen O. E.. (2011). Cathelicidin is involved in the intracellular killing of mycobacteria in macrophages. Cell. Microbiol. 13, 1601–1617. doi: 10.1111/j.1462-5822.2011.01644.x, PMID: 21790937

[ref25] Svensson L. Baumgarten M. Morgelin M. Shannon O. (2014). Platelet activation by *Streptococcus pyogenes* leads to entrapment in platelet aggregates, from which bacteria subsequently escape. Infect. Immun. 82, 4307–4314. doi: 10.1128/IAI.02020-14, PMID: 25069984 PMC4187850

[ref26] Tenland E. Krishnan N. Ronnholm A. Kalsum S. Puthia M. Morgelin M.. (2018). A novel derivative of the fungal antimicrobial peptide plectasin is active against *Mycobacterium tuberculosis*. Tuberculosis 113, 231–238. doi: 10.1016/j.tube.2018.10.008, PMID: 30514507 PMC6289163

[ref27] Tenland E. Pochert A. Krishnan N. Umashankar Rao K. Kalsum S. Braun K.. (2019). Effective delivery of the anti-mycobacterial peptide NZX in mesoporous silica nanoparticles. PLoS One 14:e0212858. doi: 10.1371/journal.pone.0212858, PMID: 30807612 PMC6391042

[ref28] WHO (2024a). WHO bacterial priority pathogens list, 2024. Geneva: World Health Organization.

[ref29] WHO (2024b). Global tuberculosis report 2024. Geneva: World Health Organization.

[ref30] Xiong Y. Q. Hady W. A. Deslandes A. Rey A. Fraisse L. Kristensen H. H.. (2011). Efficacy of NZ2114, a novel plectasin-derived cationic antimicrobial peptide antibiotic, in experimental endocarditis due to methicillin-resistant *Staphylococcus aureus*. Antimicrob. Agents Chemother. 55, 5325–5330. doi: 10.1128/AAC.00453-11, PMID: 21859940 PMC3195053

[ref31] Zhang Y. Teng D. Mao R. Wang X. Xi D. Hu X.. (2014). High expression of a plectasin-derived peptide NZ2114 in Pichia pastoris and its pharmacodynamics, postantibiotic and synergy against *Staphylococcus aureus*. Appl. Microbiol. Biotechnol. 98, 681–694. doi: 10.1007/s00253-013-4881-2, PMID: 23624708

