# Supplementary figures and images for: Antimycobacterial activity of the plectasin derivative NZ2114
Source: Front Microbiol. 2025 Jun 26;16:1613241. doi: 10.3389/fmicb.2025.1613241 (PMC12241124; doi:10.3389/fmicb.2025.1613241)

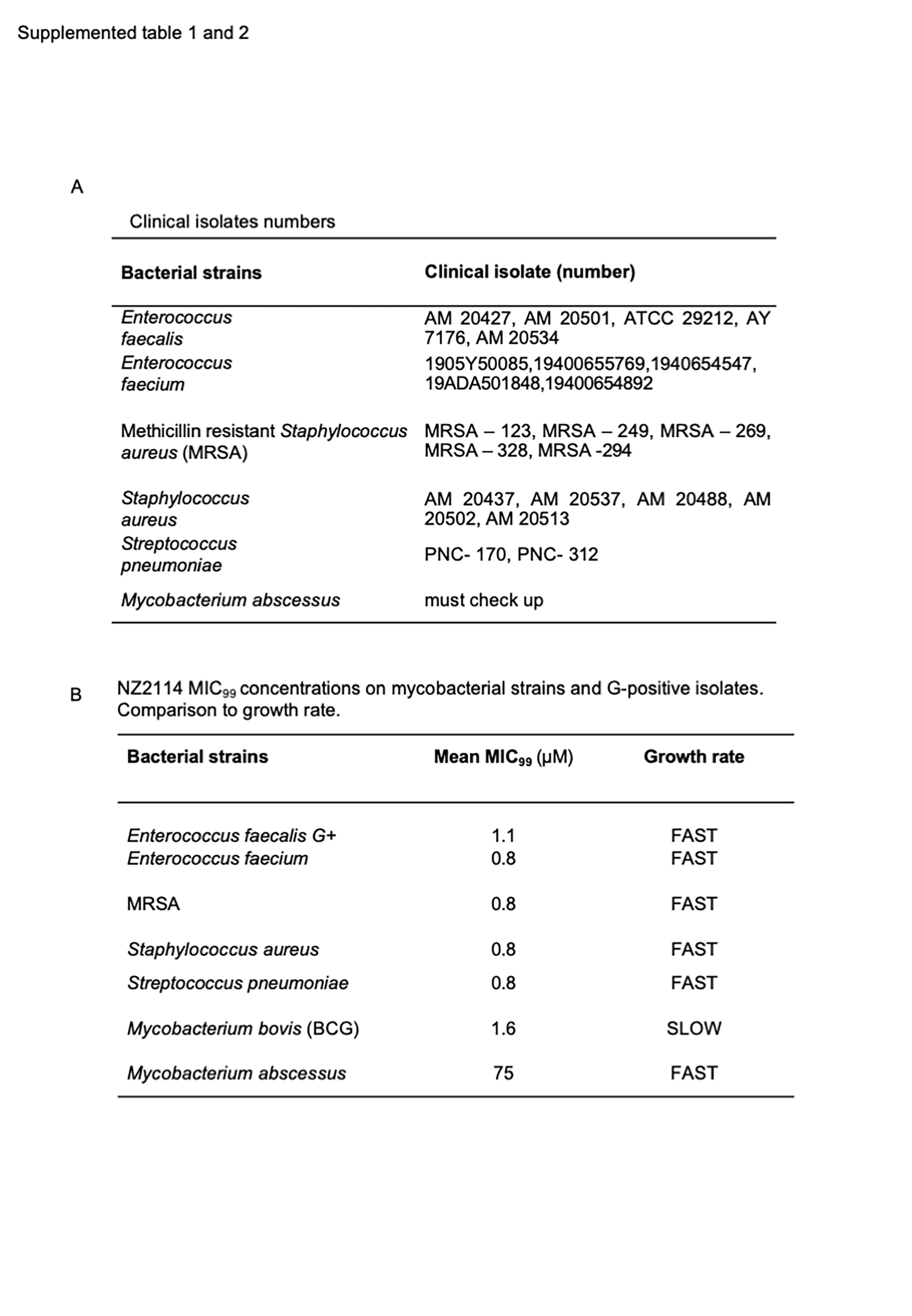

Supplement: Supplementary file 1 [file Image_2.TIFF]
